# The Association of Dietary Energy Density and Body Composition Components in a Sample of Iranian Adults

**DOI:** 10.3389/fnut.2021.751148

**Published:** 2021-10-29

**Authors:** Elham Bazshahi, Sanaz Pourreza, Hossein Imani, Leila Azadbakht, Mojdeh Ebaditabar, Samira Davarzani, Nadia Babaei, Sina Naghshi, Kurosh Djafarian, Sakineh Shab-Bidar

**Affiliations:** ^1^Department of Community Nutrition, School of Nutritional Sciences and Dietetics, Tehran University of Medical Sciences (TUMS), Tehran, Iran; ^2^Department of Clinical Nutrition, School of Nutritional Sciences and Dietetics, Tehran University of Medical Sciences (TUMS), Tehran, Iran; ^3^Diabetes Research Center, Endocrinology and Metabolism Clinical Sciences Institute, Tehran University of Medical Sciences, Tehran, Iran; ^4^Food Security Research Center, School of Nutrition and Food Science, Isfahan University of Medical Sciences, Isfahan, Iran

**Keywords:** dietary energy density, obesity, waist circumference, body composition, adults

## Abstract

**Background:** We aimed to investigate the association between the energy density (ED) of diet and body composition components in Iranian adults.

**Methods:** We conducted a cross-sectional study on 267 adults in Tehran. We obtained ED (kcal/g) using the two most common methods: ED1, ED from foods only with the exclusion of all beverages and ED2, from foods and all beverages. Body composition was measured using a multifrequency bio-impedance analysis. To find a strong association, we used both the linear and binary regression analysis in the three adjusted models.

**Results:** The mean of ED1 and ED2 was 1.34 ± 0.23 and 0.89 ± 0.20 kcal/g, respectively. Increasing the ED of diet in both methods was associated with a high intake of dietary fat, of saturated fatty acid (SFA), of monounsaturated fatty acid (MUFA), of polyunsaturated fatty acid (PUFA), of oleic and linoleic acids, accompanied by a low intake of fruits, vegetables, and some vitamins and minerals. There was a significant positive relationship between fat-free mass index (FFMI) and ED1 (β = 4.44, *p* = 0.02). However, we found no significant association between the consumption of ED1 and fat mass index (FMI) (0.28; 95% CI 0.08, 0.98; *p* = 0.07), and abdominal obesity (0.91; 95% CI 0.43, 1.94; *p* = 0.82). Also, ED2 had no association with FMI (0.86; 95% CI 0.26, 2.80; *p* = 0.81) and abdominal obesity (0.78; 95% CI 0.35, 1.72; *p* = 0.54). No significant associations were found between ED and other anthropometric indices and body composition components after considering the confounders.

**Conclusion:** This study supports the positive association between ED and poor dietary quality. However, our findings did not show significant associations of dietary energy density (DED) with anthropometric indices and body composition components. Further well-designed studies are required to investigate the exact link between DED and body composition.

## Introduction

Measuring body composition, especially energy needs and nutritional status, is very important due to its important clinical applications, particularly in the assessment and management of obesity and its related comorbidities (1). Researchers suggest that abdominal obesity is a better indicator of the risk of chronic diseases such as metabolic syndrome, type 2 diabetes, cardiovascular disease, and mortality (2). The prevalence of abdominal obesity is increasing at an alarming rate around the world (3). Indicators such as percentage body fat (PBF), fat mass index (FMI), and fat-free mass index (FFMI) are more stable in obesity than in body mass index (BMI) or body weight indices (4).

Several studies have examined the role of dietary patterns, food groups, and single nutrients in obesity (5). Recently, much attention has been paid to the energy density (ED) of diet and the etiology of obesity (6). The ED of food can be defined as the amount of metabolizable energy per unit weight of a food (kJ/g or kcal/g) (7) and is obtained by the macronutrient and moisture content of the food. Fat [2.15 kJ/g (9 kcal/g)] and water (0 kJ/g) as most and least energy-dense nutrients are the primary determinants of ED. Dietary energy density (DED) can be defined as the ED of a total diet. No consensus has been reached on the appropriate method for calculating the ED of the diet (8). However, the most commonly used method is to calculate solid foods only, which is believed to better define DED (8–10). In Iran, also among various studies, which have examined the role of ED, the only method used for calculating DED has excluded beverages and considers only solid foods (11–14).

It is often stated that a diet with high ED consumption is significantly associated with a higher risk of overweight and obesity (14). Yin found that ED was positively associated with body composition among Chinese adults (15). This relationship was noted not only in cross-sectional (10, 16) and prospective studies (17, 18) but also in clinical trials (19, 20). In contrast, other studies did not show any significant relationship (21–23).

Previous studies on this topic in the Iranian population have been performed only on women (12, 14, 24, 25) and in a specific age range (11, 25, 26). On the other hand, these studies examined the association between DED and general obesity and did not consider the relationship for the other components of body composition. Therefore, the main aim of this study was to assess the association between DED and abdominal obesity and FMI among Iranian adults.

## Subjects and Methods

### Study Population

Based on the previously calculated correlation coefficient between energy intake and FMI (*r* = 0.18) (15), our target number of participants was 185 ($Z_{1-\frac{\alpha }{2}}$$+Z_{1-\beta }\times \sqrt{1-r^{2}}$/*r*). However, to replace patients who were excluded due to under- or over-reported food intake, we continued sampling up to enrollement of 276 individuals. This study is a cross-sectional study on apparently healthy adults in Tehran, who had entered the study by a simple and convenience sampling method. Individuals involved in this study are based on the following inclusion criteria: healthy people who want to participate in this study, both sexes within the age range of 18–59 years, with no history of diabetes, cardiovascular disease or cancer, no pregnant and lactating women, no regular use of a special supplement or drug (slimming, hormonal, sedative, supplements containing thermogenic substances such as caffeine and green tea, linoleic conjugated acid), and the lack of a special diet. After excluding the participants who misreported their daily energy intake, the final analysis was conducted on 267 participants. Participation in this study was completely voluntary and, at the beginning of the study, a consent form was obtained from all participants of the ethical committee of the Tehran University of Medical Sciences (Ethics No.: IR.TUMS.VCR.REC.1396.4085).

### Assessment of Dietary Intake

Nutritional status of individuals was assessed by a 168-item food frequency questionnaire (FFQ). The reliability and validity of the FFQ for the food group intake have been assessed and found to be acceptable (27). Respondents were asked by trained dietitians to rate their frequency of use in the past year. Depending on the type of food consumption, the frequency of consumption per day, week, and month was questioned. The values for each food item were converted to grams by individuals using the household measures book manual. Also, we calculated the daily nutrient intake for each participant according to the analysis of nutrient contents of all foods using the Nutritionist IV software. For common foods, the approximate proportion of consumption is calculated based on the US Department of Agriculture's national nutrient data bank. The nutrients examined in this study were selected mainly for assessing dietary intake comprehensively while considering the current dietary intake patterns in an Iranian (28).

### Calculation of Dietary Energy Density

To estimate the ED of the diet, the reported amounts of energy (kcal/day) received from foods are divided by total weight of foods (g/day) consumed per day. Because there is currently no standard way to calculate ED, researchers each uses their methods. In this study, we obtained ED using the two most common methods (8, 29): (ED1) ED from foods only (solid, semisolid, and liquid), with the exclusion of all beverages and (ED2) ED from foods and all beverages (carbonated drinks, fruit juices and fruit-flavored drinks, milk, tea, and coffee).

### Anthropometric Measurements

Anthropometric measures, including weight, height, waist, and hip circumference (HC), were measured. The height of participants without shoes was measured by a stadiometer with a sensitivity of 0.1 cm (Seca, Hamburg, Germany) and the weight using a digital scale (808 Seca, Hamburg, Germany) with an accuracy of 0.1 kg with light clothing (without coat and raincoat). BMI was calculated by dividing the weight in kilograms by the square of height (kg/m^2^). WHO classification is commonly used to classify BMI (BMI ≤ 18.5 was defined as low weight, 18.5–24.9 favorable weight, 25–29.9 overweight, and ≥30 obese). Waist circumference (WC) was measured between the lower ribs and the iliac crest, exhaled. HC with a tape measure and the waist to hip ratio (WHR) was calculated as WC divided by HC for each individual. Abdominal obesity was defined as WC ≥102 cm (40 inches) for men and ≥88 cm (35 inches) for women, WHR > 0.9 for men, and >0.85 for women, and waist to height ratio (WHtR) greater than 0/5 (30).

The bioelectric impedance analysis was used to measure body composition components with a commercially available body analyzer (InBody 720, Biospace, Tokyo, Japan). Subjects were hydrated with two glasses of fluids before the measurement. For an accurate measurement, people were advised not to have moderate to vigorous physical activity for 1–2 h before the analysis and to empty their bladder before the measurement. Also, during the measurement, shoes and socks were removed and clothes were reduced as much as possible. The system provided FMI, FFMI, PBF, total body fat, visceral fat mass, and abdominal fat mass. FMI and FFMI were calculated using the following formulas:


$$\begin{array}{l} \text{ FMI = }(\text{weight}\times \;\%\text{BF}){\text{/height}}^{2}\\ \text{FFMI = }[\text{weight}-(\text{weight}\times \;\%\text{BF})]{\text{/height}}^{2} \end{array}$$

### Assessment of Other Variables

According to the inclusion and exclusion criteria, participants were recruited and interviewed to collect general demographic information, including age, marital status (single or married) and lifestyle (alone or with someone), education (under diploma, diploma, or educated), occupation (employee or unemployed), smoking (smoker or non-smoker), disease status (yes or no), supplementation, and medication use (yes or no), using a public information questionnaire. The International Physical Questionnaire (IPAQ) was used to evaluate physical activity (metabolic equivalent; MET.h/day) (31). According to the IPAQ criteria, data were recorded regarding vigorous and moderate activity and walking, for at least 10 min/day in the last 7 days. The duration and frequency of activity days were multiplied by the MET task value of an activity to calculate an activity. Total physical activity per week was used to calculate the sum of the scores and categorized into three groups: low, moderate, and high. Also, IPAQ was computed for a continuous score and reported as MET-min/week. Participants were asked to recall all their intense and moderate activities last week, along with time taken to complete them. Then, the intensity of each activity (MET) was multiplied by the time it was performed, and finally, these values were added together to determine the value of MET.h/day. Participants were then classified into no or low physical activity, and moderate and high physical activity (32, 33).

### Statistical Analyses

Participants were divided based on the tertiles of ED1 and ED2. To compare the general characteristics, anthropometric measurements, body composition, and dietary intake across the tertiles of DEDs, one-way ANOVA and chi-squared tests were used for continuous and categorical variables, respectively. Analysis of covariance (ANCOVA) was used to compare dietary intake among the tertiles of DED by adjusting age, gender, education, occupation, smoking, and physical activity as confounding factors. Based on WHO guidelines, we considered BMI 25–29.9 kg/m^2^ to classify overweight, BMI ≥ 30 kg/m^2^ as obese, WC ≥ 102 cm for men and ≥88 cm for women, and WHR > 0.9 for men and >0.85 for women were used as the markers of abdominal obesity (30). Odds ratios (OR) and 95% CIs were obtained using logistic regression to determine the relationship of ED1 and ED2 with the risk of body composition components including a dichotomous outcome (yes or no) and ED1 and ED2 as exposures. FMI (≥21.9), and FFMI (≥47.8) and PBF (≥30.8) were classified into high and low levels using the median.

The risk was reported in the three models (model I: crude; model II: adjusted for age and sex; and model III: adjusted for age, sex, marital status, menopause, physical activity, education, occupation, smoking status, chronic disease, and supplementation). To determine the contribution of anthropometric measurements and body composition components with ED1 and ED2, we used multiple linear regression models (model I: crude; model II: adjusted for age and sex; and model III: adjusted for age, sex, marital status, menopause, physical activity, education, occupation, smoking status, chronic disease, and supplementation). All statistical analyses were performed using the Statistical Package for Social Sciences (version 26; SPSS, Inc., Chicago, IL, USA). The value of *p* < 0.05 was considered as a statistical significance level.

## Results

A total of 267 men and women aged 18–59 participated in this cross-sectional study. General characteristics and anthropometric measurements of the participants across the tertiles of ED1 and ED2 are presented in Table 1. More than half of the participants were women, married, educated, non-smokers, employed, and living with someone. Nine percent of participants had underlying diseases (diabetes, hypertension, or hyperlipidemia). The mean of ED1 and ED2 were 1.34 ± 0.23 and 0.89 ± 0.20, respectively. The mean age of participants was 36.5 ± 13.1. Participants in the highest tertile were significantly older than those in the lowest tertile of ED1 (*p* = 0.002, *p* < 0.001) and ED2 (*p* < 0.001, *p* < 0.001). They also had higher BMI in comparison to those in the lowest tertile of ED1 (*p* = 0.01, *p* = 0.006) and ED2 (*p* = 0.002, *p* < 0.001). For ED2, WC (*p* = 0.04, *p* = 0.01) and HC (*p* = 0.02, *p* = 0.009) were significantly different across the tertiles. Meanwhile, WC and HC increased from tertiles 1 to 3 of ED1 (*p* = 0.04, *p* = 0.02), and WHR increased from the first to the last tertile of ED2 (*p* = 0.03), none of them were significantly different (*p* = 0.12, *p* = 0.07, and *p* = 0.08, respectively). FMI did not differ significantly according to the tertiles of ED1 (*p* = 0.07) and ED2 (*p* = 0.06); however, an increasing trend was detected from the first to the last tertile of both ED1 (*p* = 0.03) and ED2 (*p* = 0.02). Although energy intake did not have a statistical difference between the tertiles of ED1, it had an increasing trend from the lowest to the highest tertile (*p* = 0.07, *p* = 0.02). Across the tertiles of ED2, energy intake was significantly different (*p* = 0.009, *p* = 0.01). The frequency of obesity during the tertiles of ED2 and abdominal obesity through the tertiles of ED1 were significantly different (*p* = 0.04 and *p* = 0.01, respectively). Among the demographic characteristics, marital status was significantly different among the tertiles of ED1 (*p*= <0.001) and ED2 (*p* < 0.001).

**Table 1:** General characteristics of participants by tertiles of the dietary energy density (DED) in a sample of Iranian adults.

	**Tertiles of dietary energy density (ED1)**								**Tertiles of dietary energy density (ED2)**							
	**T1 (*n =* 89) 0.66–1.11 kcal/g**		**T2 (*n =* 89) 1.11*–*1.56 kcal/g**		**T3 (*n =* 89) 1.56*–*2.02 kcal/g**		*P* value^*^	**P trend^†^**	**T1 (*n =* 89) 0.38*–*0.76 kcal/g**		**T2 (*n =* 89) 0.76*–*1.14 kcal/g**		**T3 (*n =* 89) 1.14*–*1.52 kcal/g**		*P* value^*^	**P trend^†^**
	**Mean**	**SD**	**Mean**	**SD**	**Mean**	**SD**			**Mean**	**SD**	**Mean**	**SD**	**Mean**	**SD**		
Age (year)	33.6	12.4	35.7	11.8	40.3	14.2	0.002	<0.001	32.0	12.2	35.7	12.1	41.9	13.1	<0.001	<0.001
Weight (kg)	70.6	14.9	72.2	16.2	74.8	16.2	0.20	0.07	69.7	17.1	73.6	15.0	74.3	15.1	0.10	0.05
Height (cm)	168	9.57	168	10.4	167	9.64	0.66	0.40	168	10.0	169	10.1	166	9.30	0.13	0.09
BMI (kg/m2)	24.7	4.38	25.2	4.06	26.7	5.33	0.01	0.006	24.2	4.43	25.7	4.50	26.7	4.81	0.002	<0.001
WC (cm)	87.7	12.0	89.2	11.9	91.5	13.4	0.12	0.04	87.1	13.2	89.5	11.4	91.8	12.5	0.04	0.01
HC (cm)	97.6	7.18	98.4	7.35	100	8.14	0.07	0.02	97	7.91	99.2	7.27	99.9	7.41	0.02	0.009
WHR	0.89	0.06	0.90	0.06	0.91	0.06	0.29	0.12	0.89	0.06	0.90	0.06	0.91	0.06	0.08	0.03
WHtR	0.52	0.06	0.53	0.06	0.54	0.08	0.03	0.01	0.51	0.06	0.53	0.06	0.55	0.07	0.03	0.001
FMI (kg/m^2^)	21.3	8.68	21.5	7.46	24.2	11.4	0.07	0.03	20.9	9.17	22.1	9.23	24.1	9.68	0.06	0.02
FFMI (kg/m^2^)	49.2	11.6	50.7	13.3	50.5	11.4	0.67	0.48	48.8	12.8	51.5	12.8	50.1	10.5	0.35	0.47
PBF	29.9	9.53	30.0	7.87	31.5	10.5	0.42	0.25	29.6	9.23	30.0	9.84	31.8	8.96	0.26	0.12
Energy intake (kcal/d)	2,433	734	2,262	766	2,190	660	0.07	0.02	2,382	695	2,401	710	2,102	742	0.009	0.01
Obese (%)^‡^	11.2		11.2		22.5		0.05		10.1		12.4		22.5		0.04	
Overweight (%) ^§^	27.0		24.7		29.2		0.10		22.5		30.3		28.1		0.06	
Abdominal obesity (%) ||	23.8		15.2		35.8		0.01		21.0		24.7		29.5		0.46	
**Gender (%)**																
Male	44.9		47.2		39.3		0.55		39.3		47.2		44.9		0.55	
Female	55.1		52.8		60.7				60.7		52.8		55.1			
**Education (%)**																
Under diploma	4.5		5.6		12.4		0.26		5.6		7.9		10.1		0.69	
Diploma	15.7		19.1		20.2				16.9		16.9		21.3			
Educated	78.7		75.3		67.4				77.5		75.3		68.5			
**Occupation (%)**																
Employee	55.1		59.6		44.9		0.13		44.9		57.3		57.3		0.16	
Unemployed	44.9		40.4		55.1				55.1		42.7		42.7			
**Marital status (%)**																
Single	56.8		43.8		28.1		<0.001		62.5		44.9		21.3		<0.001	
Married	43.2		56.2		71.9				37.5		55.1		78.7			
**Menopause status**																
Yes	7.9		9.0		23.6		0.01		10.1		11.2		19.1		0.19	
No	47.2		43.8		37.1				50.6		41.6		36.0			
**Disease status**																
Yes	6.7		10.1		10.2		65.0		2.2		10.2		14.6		0.01	
No	93.3		89.9		89.8				97.8		89.8		85.4			
**Lifestyle (%)**																
Alone	7.9		7.9		11.2		0.66		9.0		12.4		5.6		0.29	
With someone	92.1		92.1		88.8				91.0		87.6		94.4			
**Smoking (%)**																
Smoker	13.5		12.4		14.6		0.90		6.7		16.9		16.9		0.07	
No smoker	86.5		87.6		85.4				93.3		83.1		83.1			
**Physical activity (%)**																
Low	40.4		39.3		34.8		0.73		46.1		34.8		33.7		0.08	
Moderate	40.4		43.8		40.4				30.3		43.8		50.6			
High	19.1		16.9		24.7				23.6		21.3		15.7			

*ED1, energy density from foods only; ED2, energy density from foods and all beverages; SD, standard deviation; BMI, body mass index; WC, waist circumference; HC, hip circumference; WHR, waist to hip ratio; FMI, fat mass index = (weight × PBF)/height; FFMI, fat-free mass index = [weight – (weight × PBF)]/height; PBF, percentage body fat calculated using sex-specific equations. Data are presented as means and SD*.

* *p-values result from ANOVA for quantitative variables and χ2 test for qualitative variables*.

† *p-values for trend analysis by using linear regression*.

‡ *Obesity: BMI ≥30.0 kg/m^2^*.

§ *Overweight: BMI 25–29.9 kg/m^2^. ||Abdominal obesity: defined as WC≥102 cm for men and ≥88 cm for women. p-value is considered significant at <0.05*.

Table 2 presents the dietary intake of nutrients and food groups among the tertiles of DED. There were significant differences in total energy intake, fat, saturated fatty acid (SFA), monounsaturated fatty acid (MUFA), polyunsaturated fatty acid (PUFA), oleic acid, linoleic and linolenic acids, soluble and insoluble fiber consumption among the tertiles of ED1. All of them remained significant after adjusting for age, gender, education, occupation, physical activity, and smoking as covariates. Participants in the highest tertile had more total energy and fat intake (*p* = 0.02 and *p* < 0.001, respectively), and a lower intake of dietary fiber (*p* < 0.001). Among vitamins and minerals, significant differences were observed for potassium (K), magnesium (Mg), calcium (Ca), vitamin A, vitamin E, folate, vitamins B5 and B6, vitamin C, and biotin. Only Ca intake was not significant across the tertiles of DED after an adjustment for covariates. Moreover, sugar consumption was significantly different across the tertiles of ED1 (*p* < 0.001). Individuals of the last tertile had a lower amount of sugar consumption. Among the main food groups, the differences were significant for fruits, vegetables, and dairy (*p* < 0.001, *p* < 0.001, and *p* = 0.01, respectively).

**Table 2:** Dietary intake of Iranian adults by tertiles of DED.

	**Tertiles of dietary energy density (ED1)**								**Tertiles of dietary energy density (ED2)**							
	**T1 (*n =* 89) 0.66*–*1.11 kcal/g**		**T2 (*n =* 89) 1.11*–*1.56 kcal/g**		**T3 (*n =* 89) 1.56*–*2.02 kcal/g**		P trend^*^	***P* value^†^**	**T1 (*n =* 89) 0.38*–*0.76 kcal/g**		**T2 (*n =* 89) 0.76*–*1.14 kcal/g**		**T3 (*n =* 89) 1.14*–*1.52 kcal/g**		P trend^*^	***P* value^†^**
	**Mean**	**SD**	**Mean**	**SD**	**Mean**	**SD**			**Mean**	**SD**	**Mean**	**SD**	**Mean**	**SD**		
**Nutrients**																
Total energy (kcal/d)	2,190	660	2,262	766	2,433	734	0.02	0.01	2,102	742	2,401	710	2,382	695	0.01	<0.001
Carbohydrate (g/d)	321	100	326	120	334	120	0.45	0.33	309	118	346	110	326	110	0.31	0.003
Protein (g/d)	89.1	33.5	85.3	40.8	88.3	30.5	0.89	0.68	79.9	29.8	96.9	41.8	85.9	30.6	0.24	0.001
Fat (g/d)	67.0	26.8	74	27.6	87	33.4	<0.001	<0.001	65.8	27.4	75.9	27.4	86.4	33.1	<0.001	<0.001
SFA (g/d)	20.0	0.14	22.2	8.70	27.2	11.8	<0.001	<0.001	20.2	9.75	23.6	9.95	25.6	10.8	<0.001	0.001
MUFA (g/d)	19.3	8.67	22.0	10.0	26.7	11.7	<0.001	<0.001	19.1	8.71	22.4	10	26.4	11.8	<0.001	<0.001
PUFA (g/d)	14.0	7.28	15.4	8.18	18.8	10.1	<0.001	0.002	13.8	7.78	14.7	6.50	19.7	10.6	<0.001	<0.001
n9-oleic (g/d)	17.2	8.77	18.8	8.58	22.1	10.7	0.001	0.002	16.1	7.61	19.2	8.82	22.7	10.9	<0.001	<0.001
n6-linoleic (g/d)	11.5	6.74	13.3	7.71	16.2	9.46	<0.001	0.002	11.6	7.35	12.3	5.98	17.1	9.89	<0.001	<0.001
n3-linolenic (g/d)	0.11	0.08	0.12	0.08	0.16	0.13	<0.001	0.001	0.12	0.09	0.13	0.10	0.14	0.11	0.15	0.15
EPA (g/d)	0.03	0.05	0.03	0.03	0.02	0.03	0.16	0.34	0.03	0.05	0.03	0.03	0.02	0.03	0.1	0.32
DHA (g/d)	0.08	0.12	0.07	0.08	0.06	0.07	0.16	0.34	0.08	0.13	0.07	0.08	0.06	0.07	0.1	0.32
Cholesterol (mg/d)	309	240	275	198	280	137	0.32	0.18	263	148	326	236	276	192	0.67	0.18
Dietary fiber (g/d)	18.1	6.56	15.1	6.75	13.5	5.01	<0.001	<0.001	15.9	7.21	16.7	6.50	14.2	5.20	0.07	0.09
Na (mg/d)	4,284	2,548	4,105	2,452	4,480	2,536	0.60	0.64	4,201	2,808	4,296	2,105	4,372	2,584	0.65	0.85
K (mg/d)	3,970	1,402	3,379	1,323	3,040	1,098	<0.001	<0.001	3,637	1,392	3,702	1,395	3,051	1,107	0.003	0.05
Ph (mg/d)	1,361	553	1,253	570	1,217	456	0.07	0.17	1,258	546	1,375	576	1,197	451	0.44	0.19
Se (mg/d)	0.03	0.02	0.04	0.03	0.04	0.02	0.21	0.45	0.03	0.2	0.04	0.03	0.04	0.03	0.15	0.14
Mg (mg/d)	306	101	274	104	256	86.4	0.001	0.01	287	106	296	103	253	83.2	0.01	0.12
Fe (mg/d)	21.5	10	21.2	10.7	19.8	6.89	0.23	0.48	18.8	8.33	23.4	11.6	20.2	6.86	0.31	0.002
Ca (mg/d)	1,103	526	950	454	923	374	0.009	0.05	1,017	521	1,069	485	889	343	0.06	0.16
Zn (mg/d)	9.85	4.01	9.19	4.42	9.13	3.25	0.21	0.35	8.73	3.58	10.3	4.65	9.12	3.26	0.5	0.01
Vitamin A (mg/d)	1,723	1,294	1,206	676	1,090	717	<0.001	<0.001	1,398	1,191	1,391	821	1,230	875	0.25	0.85
Vitamin E (mg/d)	4.79	2.77	4.20	2.49	3.76	1.79	0.004	0.02	4.07	2.31	4.7	3.0	3.98	1.62	0.8	0.11
Vitamin D (mg/d)	2.46	2.07	2.12	2.33	2.14	1.80	0.31	0.43	2.38	2.14	2.63	2.53	1.73	1.27	0.03	0.02
Folate (mg/d)	347	138	290	126	274	110	<0.001	0.002	330	139	311	123	270	116	0.002	0.17
Vitamin B1 (mg/d)	1.80	0.62	1.76	0.63	1.81	0.68	0.90	0.7	1.67	0.66	1.91	0.63	1.78	0.62	0.25	0.003
Vitamin B2 (mg/d)	1.80	0.81	1.59	0.77	1.62	0.63	0.11	0.11	1.64	0.78	1.79	0.80	1.58	0.64	0.59	0.31
Vitamin B3 (mg/d)	20.98	7.27	21.1	9.45	22.3	7.91	0.25	0.3	19.0	7.74	23.4	9.07	22	7.32	0.01	<0.001
Vitamin B5 (mg/d)	6.59	2.52	5.81	2.65	5.15	1.82	<0.001	<0.001	5.89	2.59	6.23	2.51	5.43	2.10	0.19	0.33
Vitamin B6 (mg/d)	1.66	0.70	1.39	0.69	1.28	0.52	<0.001	0.001	1.38	0.61	1.57	0.69	1.37	0.65	0.92	0.05
Vitamin B12 (mg/d)	4.74	2.90	4.16	2.33	4.47	2.73	0.50	0.17	4.27	2.4	4.9	2.86	4.2	2.68	0.87	0.32
Vitamin C (mg/d)	181	82.1	128	61.7	97.8	42.7	<0.001	<0.001	143	74.4	149	82	115	55.6	0.009	0.05
Biotin (mg/d)	30.2	15.1	25.6	10.9	23.3	10	<0.001	<0.001	26.2	11.7	29	14.7	23.9	10.3	0.21	0.06
Sugar (g/d)	93.9	37.5	84.1	41.4	73	32.7	<0.001	0.009	87.9	42.4	88.1	37.7	75	32.8	0.02	0.35
**Food groups (g/d)**																
Fruits	385	193	293	188	213	123	<0.001	<0.001	293	167	344	220	255	150	0.16	0.007
Vegetables	513	257	335	162	261	134	<0.001	<0.001	431	260	368	199	311	173	<0.001	0.17
Red meat	40.2	29	43.6	35.6	50.7	46.5	0.06	0.19	33.8	25.4	54.5	44.7	46.2	38.2	0.02	0.001
White meat and fish	73.9	65.3	73.4	85.3	69.9	52.2	0.69	0.79	60.6	48.8	85.3	87.9	71.3	62.0	0.3	0.06
Grains	418	238	447	205	448	193	0.34	0.54	400	217	474	224	439	190	0.21	0.01
Dairy	525	373	449	287	409	247	0.01	0.04	503	361	501	327	380	207	0.008	0.04
Nuts	11.5	10.6	14	19	11.1	10.9	0.85	0.34	11.8	15.1	11.3	14.4	13.4	12.6	0.44	0.21
Legumes	31.6	33.6	29.9	35.9	36.7	33.8	0.32	0.49	30.1	34.2	36.4	40.3	31.6	27.6	0.76	0.55

*ED1, energy density from foods only; ED2, energy density from foods and all beverages; SD, standard deviation. Data are presented as means and SD*.

* *P values for trend result from ANOVA using linear regression*.

† *Obtained from analysis of covariance (ANCOVA) test adjusted by age, gender, education, occupation, physical activity, and smoking*.

People at the last tertile of ED2 had a greater intake of fat, SFA, MUFA, PUFA, oleic acid, linoleic and linolenic acids, sodium (Na), and nuts. Total energy intake, fat, SFA, MUFA, PUFA, oleic acid, linoleic acid, and insoluble fiber were statistically different among the tertiles of ED2. However, the consumption of carbohydrates and protein was significant only after the adjustment of covariates (*p* = 0.003 and *p* = 0.001, respectively). Statistical differences were noted for K, Mg, vitamin D, folate, vitamin B3, vitamin C, and sugar consumption. Only vitamin D and vitamin B3 remained significantly different after the adjustment. After adjusting for covariates, the intake of iron (Fe), zinc (Zn), and vitamin B1 turns out to be significantly different across the tertiles (*p* = 0.002, *p* = 0.01, and *p* = 0.003, respectively). In terms of the main food groups, the intake of vegetables, red meat, and dairy had significant differences among the tertiles of ED2 (*p* < 0.001, *p* = 0.02, and *p*= 0.008, respectively). Participants of the first tertiles consumed higher amounts of fruits and dairy and lower amounts of red meat. Unlike vegetables, the intake of fruits and grains became statistically different after adjusting for covariates (*p* = 0.007 and *p* = 0.01, respectively).

The association between DED and obesity measures is shown in Table 3. BMI (β = 2.95, *p* = 0.01), WC (β = 6.67, *p* = 0.04), and WHtR (β = 0.04, *p* = 0.03) showed significant positive associations with ED1 in the crude model. A significant positive relationship was observed between FFM and ED1 after adjusting for covariates in the first and second model (β = 4.44, *p* = 0.02) and (β = 4.19, *p* = 0.04), respectively. Also, we found a straight association between ED2 with BMI (β = 3.23, *p* = 0. 01) and WHtR (β = 0.04, *p* = 0.02). These results were not significant after controlling for covariates.

**Table 3:** Multiple regression analysis models exploring the association of DED with WC, FMI, and body composition components in a sample of Iranian adults.

	**ED1**				**ED2**			
	**β**	**R**	**SE**	***P*_**value**_**	**β**	**R**	**SE**	***P*_**value**_**
**BMI (kg/m** ^**2**^ **)**								
Model I^*^	2.95	0.02	1.22	0.01	3.23	0.02	1.37	0.01
Model II^†^	1.47	0.18	1.14	0.19	0.78	0.18	1.29	0.54
Model III^††^	0.82	0.25	1.19	0.49	0.79	0.25	1.31	0.55
**WC (cm)**								
Model I	6.67	0.01	3.29	0.04	6.38	0.01	3.69	0.08
Model II	4.17	0.17	3.07	0.17	0.64	0.17	3.49	0.85
Model III	2.61	0.24	3.23	0.41	3.23	0.02	5.20	0.53
**WHR**								
Model I	0.03	0.01	0.01	0.08	0.03	0.009	0.01	0.12
Model II	0.01	0.13	0.01	0.33	0.002	0.13	0.01	0.93
Model III	0.007	0.18	0.01	0.67	0.004	0.02	0.02	0.87
**WHtR**								
Model I	0.04	0.01	0.01	0.03	0.04	0.01	0.02	0.02
Model II	0.01	0.18	0.01	0.37	0.01	0.17	0.02	0.53
Model III	0.005	0.23	0.01	0.77	0.009	0.23	0.02	0.66
**FMI (kg/m** ^**2**^ **)**								
Model I	4.70	0.01	2.48	0.05	3.47	0.006	2.78	0.21
Model II	2.02	0.13	2.37	0.39	0.55	0.13	2.69	0.83
Model III	0.84	0.17	2.48	0.73	0.47	0.17	2.73	0.86
**FFMI (kg/m** ^**2**^ **)**								
Model I	3.03	0.003	3.21	0.34	2.95	0.003	3.59	0.41
Model II	4.44	0.66	1.90	0.02	1.51	0.09	3.54	0.66
Model III	4.19	0.67	2.05	0.04	1.92	–0.03	4.90	0.69
**PBF (%)**								
Model I	1.87	0.002	2.48	0.44	0.10	0.001	2.77	0.53
Model II	1.5	0.15	2.34	0.52	0.53	0.42	2.17	0.80
Model III	0.64	–0.04	3.60	0.85	0.34	0.43	2.23	0.87

*ED1, energy density from foods only; ED2, energy density from foods and all beverages; SE, standard error; BMI, body mass index; WC, waist circumference; WHR, waist to hip ratio; WHtR, waist to height ratio; PBF, percentage body fat calculated using sex-specific equations; FMI, fat mass index = (weight × PBF)/height; FFMI, fat-free mass index = [weight – (weight × PBF)]/height.Results are presented as regression coefficients (β)*.

* *Model I: crude*.

† *Model II: adjusted for age and sex*.

†† *Model III: adjusted for age, sex, marital status, menopause, physical activity, education, occupation, smoking status, chronic disease, and supplementation. p < 0.05 was considered significant*.

Odds ratios and 95% CI for body composition components in the tertile of DED are presented in Table 4. No significant relationship was observed between ED with FMI, WC and other anthropometric indices, and body composition components after the control of confounders in the first and second model. However, during ED1 tertiles, overweight in the crude model and FFM in the second model had a significant decreasing effect (*p* = 0.03) (*p* = 0.04), respectively.

**Table 4:** Multivariable-adjusted odds ratios (OR) and 95% CIs of WC, FMI, and body composition components according to tertiles of DED in a sample of Iranian adults.

	**ED1**						**ED2**					
	**T1 0.66–1.11 kcal/g**	***P*_**value**_**	**T2 1.11–1.56 kcal/g**	***P*_**value**_**	**T3 1.56–2.02 kcal/g**	***P*_**value**_**	**T1 0.38–0.76 kcal/g**	***P*_**value**_**	**T2 0.76–1.14 kcal/g**	***P*_**value**_**	**T3 1.14–1.52 kcal/g**	***P*_**value**_**
			**OR (95 % CI)**		**OR (95 % CI)**				**OR (95 % CI)**		**OR (95 % CI)**	
**Obese^‡^**												
Model I^*^	1	0.05	2.29 (1.00, 5.22)	0.04	1.00 (0.39, 2.53)	1.00	1	0.05	0.48 (0.21, 1.08)	0.07	0.38 (0.16, 0.90)	0.02
Model II^†^	1	0.36	0.56 (0.23, 1.35)	0.19	0.62 (0.25, 1.50)	0.29	1	0.54	0.67 (0.28, 1.57)	0.36	0.65 (0.26, 1.61)	0.35
Model III^††^	1	0.70	0.70 (0.26, 1.86)	0.48	0.68 (0.24, 1.92)	0.47	1	0.84	0.75 (0.29, 1.94)	0.56	0.81 (0.27, 2.41)	0.71
**Overweight ^§^**												
Model I	1	0.09	2.04 (1.05, 3.94)	0.13	1.24 (0.65, 2.36)	0.03	1	0.009	0.89 (0.47, 1.71)	0.74	0.38 (0.19, 0.74)	0.005
Model II	1	0.31	0.67 (0.34, 1.33)	0.25	0.59 (0.29, 1.19)	0.14	1	0.08	1.06 (0.53, 2.10)	0.85	0.51 (0.25, 1.06)	0.07
Model III	1	0.50	0.85 (0.39, 1.86)	0.69	0.63 (0.28, 1.40)	0.26	1	0.28	0.96 (0.45, 2.07)	0.93	0.55 (0.23, 1.27)	0.16
**Abdominal obesity ||**												
Model I	1	0.42	1.79 (0.90, 3.55)	0.29	0.57 (0.25, 1.28)	0.23	1	0.05	1.57 (0.76, 3.24)	0.45	1.23 (0.59, 2.57)	0.01
Model II	1	0.67	0.39 (0.16, 0.96)	0.40	0.79 (0.34, 1.85)	0.51	1	0.47	1.08 (0.46, 2.55)	0.85	0.81 (0.33, 1.94)	0.63
Model III	1	0.97	0.98 (0.47, 2.04)	0.96	0.91 (0.43, 1.94)	0.82	1	0.83	0.89 (0.43, 1.83)	0.75	0.78 (0.35, 1.72)	0.54
**WHR**												
Model I	1	0.52	0.76 (0.42, 1.37)	0.36	0.72 (0.40, 1.31)	0.29	1	0.05	0.75 (0.41, 1.37)	0.36	0.48 (0.26, 0.87)	0.01
Model II	1	0.92	0.88 (0.46, 1.67)	0.7	0.95 (0.5, 1.82)	0.89	1	0.68	0.98 (0.51, 1.88)	0.96	0.77 (0.39, 1.50)	0.44
Model III	1	0.87	1.13 (0.54, 2.36)	0.73	1.21 (0.57, 2.58)	0.61	1	0.99	1.03 (0.50, 2.12)	0.92	0.99 (0.46, 2.17)	0.99
**WHtR**												
Model I	1	0.07	1.97 (1.08, 3.58)	0.02	1.25 (0.69, 2.27)	0.45	1	0.003	0.60 (0.33, 1.09)	0.09	0.34 (0.18, 0.63)	0.001
Model II	1	0.56	0.79 (0.42, 1.51)	0.49	0.70 (0.36, 1.35)	0.29	1	0.22	0.83 (0.44, 1.59)	0.58	0.56 (0.28, 1.09)	0.09
Model III	1	0.79	1.03 (0.49, 2.16)	0.93	0.81 (0.37, 1.76)	0.60	1	0.36	0.80 (0.38, 1.66)	0.55	0.56 (0.25, 1.24)	0.15
**FMI (kg/m2)**												
Model I	1	0.94	0.95 (0.53, 1.72)	1.00	0.87 (0.48, 1.57)	0.76	1	0.62	0.95 (0.53, 1.72)	0.88	0.76 (0.42, 1.37)	0.36
Model II	1	0.18	0.41 (0.13, 1.21)	0.57	0.39 (0.13, 1.17)	0.24	1	84.0	0.72 (0.25, 2.1)	0.55	0.85 (0.29, 2.45)	0.76
Model III	1	0.17	0.35 (0.10, 1.19)	0.13	0.28 (0.08, 0.98)	0.07	1	0.71	0.61 (0.19, 1.98)	0.41	0.86 (0.26, 2.80)	0.81
**FFMI (kg/m2)**												
Model I	1	0.90	0.66 (0.36, 1.20)	0.88	0.66 (0.36, 1.20)	0.65	1	0.01	0.69 (0.38, 1.25)	0.22	0.42 (0.23, 0.76)	0.005
Model II	1	0.15	0.84 (0.44, 1.57)	0.1	0.90 (0.47, 1.71)	0.09	1	0.17	0.88 (0.47, 1.67)	0.71	0.55 (0.28, 1.06)	0.07
Model III	1	0.10	1.03 (0.50, 2.11)	0.09	0.97 (0.46, 2.05)	0.04	1	0.30	0.77 (0.38, 1.56)	0.48	0.54 (0.25, 1.18)	0.12
**PBF (%)**												
Model I	1	0.29	1.00 (0.55, 1.80)	0.17	1.09 (0.60, 1.96)	0.17	1	0.90	0.95 (0.53, 1.72)	0.88	0.87 (0.48, 1.57)	0.65
Model II	1	0.86	1.23 (0.59, 2.55)	0.58	1.57 (0.73, 3.24)	0.75	1	0.69	1.36 (0.65, 2.83)	0.40	1.12 (0.53, 2.35)	0.75
Model III	1	0.98	1.40 (0.64, 3.06)	0.93	1.77 (0.80, 3.91)	0.95	1	0.68	1.27 (0.56, 2.87)	0.56	1.46 (0.61, 3.47)	0.39

*ED1, energy density from foods only; ED2, energy density from foods and all beverages; WHR, waist to hip ratio; FMI, fat mass index = (weight × PBF)/height; FFMI, fat-free mass index = [weight – (weight × PBF)]/height; PBF, percentage body fat calculated using sex-specific equations. Results are presented as OR and 95% CIs*.

‡ *Obesity: BMI ≥30·0 kg/m^2^*.

* *Model I: crude*.

† *Model II: adjusted for age and sex*.

†† *Model III: adjusted for age, sex, marital status, menopause, physical activity, education, occupation, smoking status, chronic disease, and supplementation.*

§ *Overweight: BMI 25–29.9 kg/m^2^. ||Abdominal obesity: defined as WC ≥102 cm for men and ≥88 cm for women. p <0.05 was considered significant*.

## Discussion

The results of this study failed to show any significant association between DED, which allowed to calculate ED in the two most common different ways (ED1, using the foods only and ED2, which considering foods and all beverages) with abdominal obesity and body fat mass. Our findings showed an only significant positive correlation between ED1 and FFM after the control of confounders. We also found that there was no association between DED and odds of body composition components. To our knowledge, this is the first study to research the association between the DED, both with and without the inclusion of beverages, with abdominal obesity as measured by WC and FMI in the Iranian population.

Despite an increase in epidemiological studies on DED, there is still no standard method for calculating it. The most common method is to eliminate drinks. This is because it is well known that drinking has a weak and different effect on controlling and regulating appetite and satiety, and the inclusion of beverages in the DED calculation can change the interpretation of findings (34, 35). As a result, the consumption of beverages should not be ignored because the energy that individuals get from consuming drinks can affect the obesity process. That is why Johnson et al. (8) suggests that the energy received from beverages should be considered as a covariate in DED analyses.

Evidence from previous observational studies on the association between ED and body composition is conflicting. In line with our findings, Van Sluijs et al. (36) in a prospective study found no association between DED from foods only with FMI and PBF at baseline or follow-up, and the only positive association between DED and WC was at baseline but not at follow-up. Marchioni et al. (37) showed no association between the ED of foods and anthropometric variables. Also, a study using the data from the China Health and Nutrition Surveys (CHNS) showed a null link between ED, including beverages, and body composition (38). In addition, de Castro reported that ED from foods only (21) and foods and beverages (23) were not significantly related to body size, height, weight, or BMI.

Contrary to our findings, a cohort study by Sasaki et al. (39) concluded that the association between the ED of foods only and body weight gain was stronger in men with normal weight but in women, the association between ED and weight change was not statistically significant. Murakami et al. (40) reported that a lower DED from foods was positively associated with a lower prevalence of abdominal obesity (WC ≥80 cm) in women but not in men. Du et al. (41), in their multicenter prospective cohort study, observed that a diet with higher ED from foods only was not associated with weight change but was positively associated with WC change. Yin et al. (15) who explored the link between ED through five methods and body composition components among Chinese adults found that all ED definitions were positively associated to higher increases of body composition among women than in men. Correa et al. (42) also revealed that the associations between DED from foods and BMI, FMI, and fat mass percent were statistically significant in young adults aged 18–25 years. The results of these studies are the same as those found in the published systematic reviews and meta-analyses, which have concluded that there is a significant relationship between the ED of food and BMI (43, 44). In addition, a systematic review in children and adolescents as well as in adult, also supports a relationship between ED and body weight (45). Similarly, another cohort study demonstrated that weight gain was positively related to greater DED during a 6-year follow-up in overweight subjects (46). Therefore, these studies support the hypothesis that reducing DED can be a useful strategy for controlling body weight. These discrepant findings may be explained by a series of methodological issues like ethnic and socioeconomic differences and by the method of dietary assessment in studies. All self-reported dietary assessment methods are subject to both random and systematic measurement errors (47). Given a day-to-day variation in the dietary intake of individuals, the estimates of dietary intake derived from a dietary record used in some studies (40, 48) unlikely represent the usual intake of individuals. For this reason, we used a validated FFQ for dietary assessment, which reflects an individual's long-term habitual dietary intake. Furthermore, the underreporting of energy intake during these research studies is a problem that has to be considered (21, 38). On the other hand, gender differences that affect body composition should not be ignored. Another reason for conflicting results is due to a variation within the definitions of ED. Because DED considers all foods together, it provides a general definition of diet. For this reason, DED in each population has its characteristics that can affect the final results. Therefore, in different populations with respective dietary patterns, we need more studies to find the right relationship between DED and body composition components (12). For example, in Asian populations, DED contains foods high in water (such as rice, noodles, and fish and shellfish), whereas in western countries with a lower intake of energy-dense foods (such as fats and oils, sugar, and confectionery) (10, 40, 49). However, it should be noted that the calculation methods are comparable when their food items were the same. In that case, their comparison is correct. Because the DED obtained in this study are derived from the data collected in this population, it is not expected that these EDs will be true in a population with different eating habits.

We found that lower ED was related to favorable dietary intake patterns for participants during this study, including a higher amount of fruits, vegetables, and a lower intake of total energy, carbohydrate, fat and oils, SFA, sodium, and red meat. In this regard, several studies showed that diets with high ED have more refined grains, processed food, sugars, and fats and as a result, have low fruits, vegetables, and whole grains (19, 40, 50, 51). In our research, the mean of ED1 and ED2 was 1.34 and 0.89 kcal/g, respectively. Like other studies in this area, in Iranian populations (11, 13, 14), these results were noticeably under those results shown in western studies (1.79–1.85 kcal/g) (9, 10, 48, 49, 52). This means that subjects in this study generally consume a low-energy-dense diet than *the* rating of DED definition. The diet in Middle Eastern countries is different from that in the USA, Europe, and also other parts of Asia. This could be because of higher consumption of fats and sugar in western countries vs. a higher intake of rice and traditional bread in eastern countries. It is important to note that low DED and high DED foods varied not only in the ED but also in the composition of fat, protein, carbohydrates, and grams of fiber. Therefore, it is likely that the effects observed may be due to these differences in nutritional properties between DED food and high DED food (53). Likewise, we found that lower DED was linked to a lower BMI, but FMI, FFMI, PBF, and WC were not found to differ statistically according to the tertiles of ED1 and ED2; however, an increasing trend was detected from the first to the last tertile of both ED1 and ED2. This could be related to the age of participants. Participants in the highest tertile were significantly older than the participants in the lowest tertile of ED1 and ED2. In general, during aging, PBF increases, and FFM, lean mass, and bone mineral density decrease. Moreover, the increase in FM is distributed more specifically in the abdominal region (54).

The exact mechanisms through which low DED decreased BMI have not yet been perfectly understood, but it has been hypothesized that eating low-energy-dense foods instead of foods higher in ED might enhance satiety and lead to a significantly reduced energy intake (50). Several studies have found that a diet with higher ED is associated with poor appetite control (55) and higher body weight (48, 56). The volume of food acts as a satiety indicator. Therefore, consuming foods with high ED increases energy intake (57, 58). In several clinical and laboratory studies, the relationship between energy-dense foods with appetite stimulation and satiety has been investigated. One study measured satiety from 240 kcal of 38 common foods in the weight ranging from 38 to 625 g, which showed that the satiety index is inversely related to the weight of foods consumed. Dense foods such as chocolate and cakes were less satiety than low-density foods such as boiled potatoes and fish (59). Another study examined the relationship between ED, satiety, and food pleasantness. Low-density foods were more satiety but less palatable, whereas high-density foods were less satiety and more palatable (60, 61). A small number of body weight measuring studies reported a small amount of weight gain at the end of high DED (62–65). In the two studies, a higher energy intake was associated with lower density (66, 67). In some studies, excessive energy intake was associated with higher consumption of high-calorie beverages (68). But it is not yet clear whether density will lead to weight gain or overeating (60).

This study has several strengths and limitations. In this study, abdominal obesity was evaluated in addition to WC, including WHR and WHtR. However, the general statistics of general and abdominal obesity were low. So, it is hard to find a connection. Despite finding a more accurate relationship, we performed our results based on different analyses (linear and logistic regression and comparison of means). Moreover, the adjustment for important confounders was the strength of this study. Furthermore, we use FFQ to assess dietary intake. Also, several limitations of this study warrant mention. Firstly, the cross-sectional nature of the study does not permit the assessment of causality owing to the uncertain temporality of the association. Only a prospective study would provide a better understanding of the relationship between DED and obesity. On the other hand, our sample size in this study was not sufficient for a definite conclusion. Therefore, our findings based on the small sample size in this study may not be generalizable to society.

## Conclusion

In summary, the results of this study did not show a significant relationship between ED with abdominal obesity, FMI, and other indices and body composition components in both Iranian men and women. However, our findings confirmed the profound effects that a higher energy-dense diet was associated with lower quality. Due to the association between poor quality diets and chronic diseases such as cardiovascular disease, diabetes, and obesity, high ED diets may be a risk factor. Further well-designed studies are required to investigate the exact link between DED and body composition.

## Data Availability Statement

The raw data supporting the conclusions of this article will be made available by the authors, without undue reservation.

## Ethics Statement

The studies involving human participants were reviewed and approved by Ethical Committee of the Tehran University of Medical Sciences (Ethic Number: IR.TUMS.VCR.REC.1396.4085). The patients/participants provided their written informed consent to participate in this study.

## Author Contributions

EB and SS-B contributed to the design of this research. NB, SD, and ME contributed to the acquisition and interpretation of the data. EB did analysis. EB, SP, and SN drafted the manuscript. KD, SS-B, and LA critically revised the manuscript. SS-B supervised the study. All authors read and approved the final manuscript.

## Conflict of Interest

The authors declare that the research was conducted in the absence of any commercial or financial relationships that could be construed as a potential conflict of interest. The reviewer YJ declared a shared affiliation with the authors to the handling editor at the time of the review.

## Publisher's Note

All claims expressed in this article are solely those of the authors and do not necessarily represent those of their affiliated organizations, or those of the publisher, the editors and the reviewers. Any product that may be evaluated in this article, or claim that may be made by its manufacturer, is not guaranteed or endorsed by the publisher.
